# Supplementary material for: Individual Deviation-Based Functional Hypergraph for Identifying Subtypes of Autism Spectrum Disorder
Source: Brain Sci. 2024 Jul 24;14(8):738. doi: 10.3390/brainsci14080738 (PMC11352392; doi:10.3390/brainsci14080738)
Supplement: Supplementary file 1 [file brainsci-14-00738-s001.zip › brainsci-3083091-supplementary.pdf]

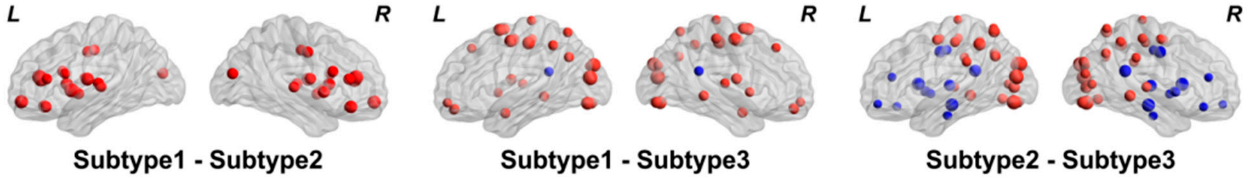

Figure S1. Comparisons of ALFF between ASD subtypes. Group differences were calculated using two-sample *t*-test. Brain regions with FDR $q < 0.05$ were visualized.

Since subtype 4 showed no significant difference across the whole brain, it tends to favor the TD group. Thus, this experimental analysis did not include it. The two-sample *t*-test by which we compared the ALFF across the other ASD subtypes revealed multiple brain areas showing a significant main group effect. Specifically, differences were found in the insula, hippocampus, parahippocampus, and some subcortical nuclei (e.g., caudate and putamen) between subtype 1 and subtype 2 (FDR $q < 0.05$, Figure S1). Compared to subtype 3, subtype 1 showed increased ALFF throughout the brain but only decreased in posterior cingulate gyrus (FDR $q < 0.05$, Figure S1), significantly increased in the cuneus, parietal, and occipital regions, and decreased in the orbitofrontal, insula, cingulate, hippocampus, parahippocampus, and some subcortical nuclei (e.g., caudate, putamen, pallidum, and thalamus) between subtype 2 and subtype 3 (FDR $q < 0.05$, Figure S1).
